# Supplementary material for: Surface-to-volume scaling and aspect ratio preservation in rod-shaped bacteria
Source: eLife. 2019 Aug 28;8:e47033. doi: 10.7554/eLife.47033 (PMC6742476; doi:10.7554/eLife.47033)
Supplement: Supplementary file 1. [file elife-47033-supp1.docx]

|  | Perturbations  or Conditions | Number of cells per  experiment or condition | Number of experiments  or conditions | Figure |
| --- | --- | --- | --- | --- |
|  |  |  |  |  |
| **Nonejuie *et al.*** | Antibiotics | ≥ 50 | 44 | Figure 1 A |
|  |  | ≥ 30 for elongated cell phenotype |  | Figure 4 B |
| **Si *et al.*** | DnaA knockdown | 5205 - 9695 | 4 | Figure 1 A, B, C Figure 1 - figure supplement 1 Figure 3 - figure supplement 1 A |
|  | SeqA knockdown | 4225 - 12034 | 8 |  |
|  | DiaA knockdown | ≥ 1000 | 8 |  |
|  | oriC block | 5939 - 10573 | 8 |  |
|  | Rep knockdown | 3144 - 5076 | 7 |  |
|  | SulA overexpression | 1215 - 6560 | 7 |  |
|  | MreB knockdown | 3718 - 18207 | 7 |  |
|  | FtsZ knockdown | ≥ 1000 | 8 |  |
|  | Fosfomycin | 1619 - 11170 | 7 |  |
|  | Triclosan | 4830 - 19082 | 7 |  |
|  | Hydroxyurea | 22158 - 36818 | 7 |  |
|  | Cephalexin | 2480 - 12429 | 8 |  |
|  | Chloramphenicol | 2202 - 21335 | 46 |  |
|  | Erythromycin | 5985 - 18955 | 16 |  |
|  | Rifampicin | 3458 - 10429 | 7 |  |
|  | Nutrient conditions | 2384 - 19566 | 15 |  |
| **Harris and Theriot** | Chloramphenicol | 82 | 5 | Figure 1 A |
|  | Fosfomycin | 190 | 5 |  |
| **Vadia *et al.*** | Nutrient conditions | 100 | 22 | Figure 1 A |
|  | Untreated | 100 | 3 |  |
|  | Rifampicin | 100 | 6 |  |
|  | Chloramphenicol | 100 | 5 |  |
|  | Cerulenin | 100 | 6 |  |
|  | Membrane synthesis | 100 | 21 |  |
| **Gray *et al.*** | Nutrient conditions | 1797-71488 | 1 | Figure 1 A |
| **Campos *et al.*** | Single deletion Keio | 174 - 408 | 4467 | Figure 1 A |
| **Taheri-Araghi *et al.*** | Glycerol | 14134 | 1 | Figure 2 A, B, D Figure 2 - figure supplement 1 C,D Figure 3 - figure supplement 1 F |
|  | Sorbitol | 10625 | 1 |  |
|  | Glucose + 12 a.a. | 18534 | 1 |  |
|  | Synthetic rich | 17574 | 1 |  |
|  | TSB | 18248 | 1 |  |
| **Harris and Theriot** | *C. crescentus* | 120 - 245 | 13 | Figure 1 E |
|  | *L. monocytogenes* | 113 - 185 | 7 |  |
| **Wright *et al.*** | *C. crescentus* | 250 | 1 | Figure 1 E |
| **Desmarais *et al.*** | *V. cholerae* | 1191 - 5448 | 5 | Figure 1 E |
|  | *P. aeruginosa* | 810 - 13065 | 6 |  |
| **Lopez-Garrido *et al.*** | *B. thuringiensis* | 20 - 30 | 1 | Figure 1 E |
|  | *B. pumilus* | 20 - 30 | 1 |  |
| **Ojkic *et al.*** | *B. subtilis* | 20 - 30 | 1 | Figure 1 E |
| **Carabetta *et al.*** | *B. subtilis* | 150 | 1 | Figure 1 E |
| **Quach *et al.*** | *S. aureus* | 12 - 71 | 406 | Figure 1 E |
| **F. Sato** | *H. pylori* | not reported | not reported | Figure 1 E |
| **S. Trachtenberg** | *S. ma­­­liferum* | not reported | not reported | Figure 1 E |
| **A. E. Pelling *et al.*** | *M. xanthus* | not reported | not reported | Figure 1 E |
| **S. Chattopadhyay** | *B. diminuta* | not reported | not reported | Figure 1 E |
