# Supplementary material for: Surface-to-volume scaling and aspect ratio preservation in rod-shaped bacteria
Source: eLife. 2019 Aug 28;8:e47033. doi: 10.7554/eLife.47033 (PMC6742476; doi:10.7554/eLife.47033)
Supplement: Supplementary file 2. [file elife-47033-supp2.docx]

|  | Perturbations or Conditions | Number of cells per experiment or condition | Figure |
| --- | --- | --- | --- |
| **Gray *et al.*** | *A. excentricus* | 1206 | Figure 1 E |
|  | *Anaerostipes sp.* | 1008 |  |
|  | *A. tumefaciens* | 2046 |  |
|  | *B. bacteroides* | 2138 |  |
|  | *B. diminuta* | 1232 |  |
|  | *B. megaterium* | 633 |  |
|  | *B. ovatus* | 2686 |  |
|  | *B. subtilis* | 882 |  |
|  | *B. subvibrioides* | 3951 |  |
|  | *B. thailandensis* | 580 |  |
|  | *B. theta,* cecum | 819 |  |
|  | *B. theta,* fecal matter | 815 |  |
|  | *B. theta,* GMM | 2238 |  |
|  | *B. theta,* TYG | 4766 |  |
|  | *B. xylanisolvens* | 4350 |  |
|  | *C. aerofaciens* | 2331 |  |
|  | *C. algicola* | 963 |  |
|  | *C. boltae* | 1963 |  |
|  | *C. crescentus* | 2557 |  |
|  | *C. hathewayi* | 2245 |  |
|  | *C. hutchinsonii* | 1657 |  |
|  | *C. indologenes* | 5110 |  |
|  | *C. violaceum* | 2290 |  |
|  | *E. coli,* anaerobic, GMM | 1090 |  |
|  | *E. coli,* LB | 1549 |  |
|  | *F. johnsoniae* | 1086 |  |
|  | *H. rosenbergii* | 1602 |  |
|  | *H. volcanii,* YPC | 1576 |  |
|  | *J. lividum* | 611 |  |
|  | *L. reuteri* | 853 |  |
|  | *L. sphaericus* | 1484 |  |
|  | *M. xanthus* | 1674 |  |
|  | *P. alcalifaciens* | 1719 |  |
|  | *P. distasonis* | 2399 |  |
|  | *P. polymyxa* | 1283 |  |
|  | *P. syringae* | 1731 |  |
|  | *R. intestinalis* | 2013 |  |
|  | *R. leguminosarum* | 1233 |  |
|  | *S. meliloti* | 1017 |  |
|  | *V. fischeri* | 2377 |  |
|  | *V. harveyi* | 1095 |  |
